# USP15 promotes the apoptosis of degenerative nucleus pulposus cells by suppressing the PI3K/AKT signalling pathway

**DOI:** 10.1111/jcmm.15971

**Published:** 2020-11-01

**Authors:** Bin Yu, Bin Shen, Zhaoyu Ba, Zhonghan Liu, Jing Yuan, Weidong Zhao, Desheng Wu

**Affiliations:** ^1^ Department of Spine Surgery Shanghai East Hospital Tongji University School of Medicine Shanghai China

**Keywords:** AKT, degenerative nucleus pulposus, FK506‐binding protein 5, miR‐338‐3p, ubiquitin‐specific protease 15

## Abstract

**Objectives:**

Degenerative disc disease is characterized by an enhanced breakdown of its existing nucleus pulposus (NP) matrix due to the dysregulation of matrix enzymes and factors. Ubiquitin‐specific protease 15 (USP15) is reported to be abnormal in certain human diseases. However, its role in NP degeneration remains unclear. Therefore, we aimed to explore the function of USP15 in degenerative NP cell specimens.

**Methods:**

We induced gene silencing and overexpression of USP15 in degenerative NP cells using RNA interference (RNAi) and a lentiviral vector, respectively. qRT‐PCR and Western blotting were used to determine gene and protein expression levels. Cell apoptosis was analysed via flow cytometry. Protein interaction was examined by performing a co‐immunoprecipitation assay. Furthermore, the PI3K inhibitor LY294002 and agonist IGF‐1 were used to investigate the link between USP15 and AKT in NP degeneration.

**Results:**

We found that USP15 was up‐regulated in degenerative NP cells and that its overexpression accelerated the process of apoptosis. Moreover, USP15 expression levels negatively correlated with AKT phosphorylation in degenerative NP cells. Furthermore, targeting and silencing USP15 with miR‐338‐3p and studying its interaction with FK506‐binding protein 5 (FKBP5) revealed enhancement of FKBP5 ubiquitination, indicating that USP15 is a component of the FKBP5/AKT signalling pathway in degenerative NP cells.

**Conclusions:**

Our results show that USP15 exacerbates NP degradation by deubiquitinating and stabilizing FKBP5. This in turn results in the suppression of AKT phosphorylation in degenerative NP cells. Therefore, our study provides insights into the understanding of USP15 function as a potential molecule in the network of NP degeneration.

## 1 INTRODUCTION

The nucleus pulposus (NP) is a critical, central portion of the intervertebral disc that contains a soft, gelatinous (mucoid) substance comprising proteoglycans (mainly aggrecan) and type II collagen,^1^ both of which are often down‐regulated in degenerative NP cells.^2^, ^3^, ^4^ Furthermore, the abnormal breakdown of NP is closely associated with the progression of intervertebral disc degeneration (IDD).^5^ Therefore, suppressing NP degradation has provided new insights into IDD treatment. However, the underlying molecular mechanism of NP degeneration has not yet been fully understood.

Ubiquitin‐specific carboxyl‐terminal protease 15 (USP15) is a member of ubiquitin‐specific proteases (USPs). USPs are cysteine protease deubiquitinase (DUB) enzymes that contain two highly conserved cysteine and histidine boxes in their structures. These boxes signify the catalytic core for proteasomal degradation,^6^ which include a polyubiquitin chain disassembly as well as the hydrolysis of ubiquitin‐substrate bonds. As a result, these DUBs play pivotal roles in protein homeostasis by regulating their turnover. These processes are critical in a broad range of regulatory pathways, including signal transduction, DNA damage and repair, cell‐cycle progression, apoptosis, receptor‐mediated endocytosis and signal transduction. A previous study has demonstrated that USP15, as expected, is vital for regulating the degradation of viral and cellular proteins.^7^ Moreover, USP15 overexpression inhibits the apoptosis of multiple myeloma cells.^8^ Furthermore, it has been reported that the AKT signalling pathway is a critical player in IDD.^9^ Activation of the AKT axis has been shown to contribute in alleviating NP degradation and accelerating USP15 translation as well as in activating the PI3K/AKT pathway.^10^, ^11^ However, the biological function of USP15 and its relationship with AKT remain unclear in human degenerative NP cells.

MicroRNAs (miRNAs) are a class of RNA 18‐22 nucleotides in length. They play multiple roles in various cell biological processes. It has been reported that miR‐338‐3p suppresses the migration and proliferation of gastric cancer cells.^12^ In addition, it inhibits AKT phosphorylation (p‐AKT) in gastric cancer cells.^13^ More importantly, the Targetscan database (http://www.targetscan.org) has predicted USP15 to be a target gene of miR‐338‐3p. However, the precise association between miR‐338‐3p and USP15 in human degenerative NP cells remains uninvestigated.

FK506‐binding protein 5 (FKBP5/FKBP51) is a critical regulator of stress responses, leading to the presumption of its role as a tumour suppressor.^14^ Moreover, accumulating evidence has indicated that FKBP5 down‐regulates AKT activation in vitro.^15^, ^16^ In contrast, activation of the AKT pathway has had been shown to suppress the apoptosis of human disc NP cells.^17^ Therefore, it is plausible to postulate that the negative effect of FKBP5 on AKT could lead to enhanced apoptosis of NP cells. Moreover, FKBP5 exacerbates impairments in cerebral ischaemic stroke by regulating the AKT/FOXO3 pathway.^18^ Additionally, it has been confirmed that USP49 deubiquitination stabilizes FKBP51, which in turn enhances the capability of pleckstrin homology domain leucine‐rich repeat protein phosphatases (PHLPPs) to dephosphorylate AKT,^19^ thereby, promoting apoptosis. PHLPPs are intracellular signalling molecules that have emerged as essential regulators of cellular homeostasis; their dysregulation has been associated with various pathophysiologies, including cardiovascular and degenerative diseases as well as cancer and diabetes.

USP15 is identified as a deubiquitinating enzyme and plays an essential role in dynamic protein‐protein interactions.^20^ However, the precise connection between USP15 and FKBP5 in human degenerative NP cells remains unclear.

The present study aimed to explore the role of USP15 and its potential signalling pathway in human degenerative NP cells. Our findings not only deepen the understanding of the role of USP15 but also indicate its potential signalling role in human degenerative NP cells.

## 2 MATERIALS AND METHODS

### 2.1 Human NP tissues

We conducted a research study using NP tissue specimens that were collected from a total of 18 patients admitted to the Shanghai East Hospital, Shanghai, China, from February 2015 to August 2015, who underwent lumbar spine surgery. Of them, 10 had degenerative disc disease (DDD) during discectomy and intervertebral fusion surgery [referred (mean age: 43, range: 33‐50; 6 males, 4 females)] and had donated their NP tissue samples for the study. Additionally, a total of eight age‐matched patients with lumbar vertebral fractures who underwent posterior discectomy, spinal fusion, decompression and stability procedures within 24 hours of trauma and with mild‐degenerative NP (mean age, 42; age range, 34‐48; 3 males, 5 females) also donated samples. These latter patients did not have a documented clinical history of lower back pain; therefore, their tissue samples were used as controls (named normal) in the study.

### 2.2 Ethical considerations

All participants who donated specimens for the study provided written informed consent. This study was approved by the Institutional Research Ethical Committee of Shanghai East Hospital (Approval 112). All procedures performed in the study involving human participants were in accordance with the ethical standards of the Institutional and/or National Research Committee of Shanghai East Hospital and the 1964 Helsinki Declaration and its later amendments in 2008.

### 2.3 Immunohistochemistry staining

Each of the tissue sections was flash‐frozen in liquid nitrogen and stored at −80°C until analysis. The frozen tissues were cryosectioned (7‐µm thick sections) and fixed in methanol (4%) for 30 minutes. Then, endogenous peroxidase activity was eliminated by incubating the sections with 3% H_2_O_2_ for 10 minutes. The slides were treated with Triton X‐100 and 5% goat serum for cell permeabilization and blocking, respectively. Thereafter, the tissue sections were incubated with primary anti‐collagen II (ab34712, Abcam, UK) and Aggrecan (ab36861, Abcam) antibodies for 1 hour at room temperature, followed by incubation with the anti‐goat horseradish peroxidase (HRP)‐3,3′‐diaminobenzidine secondary antibody for 30 minutes. The samples were counterstained with haematoxylin for 3 mins, and brown stained‐positive cells were visualized under a light microscope.

### 2.4 Cell culture

The primary NP cells used in this study were isolated from normal or degenerative NP tissues (n = 6) and cultured in Dulbecco's modified Eagle medium supplemented with foetal bovine serum (10%; 16000‐044, GIBCO, USA) and penicillin‐streptomycin mixture (1%, P1400‐100, Solarbio, China) at 37°C with 5% CO_2_. The AKT agonist IGF‐1 (20 ng/mL; 100‐11, Peprotech, USA), PI3K inhibitor LY294002 (10 μM, S1105, Selleck, USA) and MG132 (10 μmol/L; Selleck, USA) were dissolved in DMSO (D2650, Sigma, USA) and used for cell culturing. The sequences of the microRNA negative control (NC) and miR‐338‐3p mimic and inhibitor are listed in Supplementary File S1.

### 2.5 Silencing and overexpression of USP15

Three small interfering RNAs (siRNAs) targeting the different regions of human USP15 (NM_001252078.1) and a non‐specific siRNA (siNC) were each linked to the lentiviral vector (pLKO.1) and were used to prepare recombinant constructs. Each construct was transiently transfected into degenerative or normal NP cells using Lipofectamine 2000 (Invitrogen, USA). The sequence information of siUSP15s is listed in Supplementary Table S1.

The pLVX‐puro vector containing the full‐length USP15 (NM_001252078.1) cDNA sequence (oeUSP15) was transiently transfected into degenerative NP cells. The mock plasmid (oeNC) was transfected to act as an NC (oeNC). Analyses were conducted at 48 hours post‐transfection.

### 2.6 RNA extraction and quantitative real‐time polymerase chain reaction (qRT‐PCR)

Total RNA was extracted from degenerative and control NP tissues using the TRIzol Reagent kit (1596‐026, Invitrogen, USA) and converted to complementary DNA (cDNA) via qRT‐PCR using a cDNA synthesis kit (Fermentas, Canada). Then, real‐time detection (ABI‐7300, ABI, USA) was performed using the SYBR Green master mix (# K0223, Thermo, USA). GAPDH was used as a control for mRNAs, whereas the RNU6B (U6) gene was used as a control for miRNAs. Three replications were performed for all reactions. The primer sequences are listed in Supplementary File S2.

### 2.7 Western blotting

Total protein was extracted from degenerative and control NP tissues using the RIPA lysis buffer (JRDUN, Shanghai, China). The bicinchoninic acid assay kit (Thermo Fisher, USA) was used to measure total protein concentration. An amount of 25 μg of protein of each sample was separated using 10% sodium dodecyl sulphate‐polyacrylamide gel electrophoresis (SDS‐PAGE), followed by transferring the gels onto PVDF nitrocellulose membranes (Millipore, USA) for 12 hours. Then, the membranes were probed with the respective primary antibodies at 4°C overnight, followed by addition of appropriate HRP‐conjugated goat anti‐rabbit IgG (Beyotime, China) at 37°C for 60 minutes. Protein signals were detected using a chemiluminescence system (Tanon, China). GAPDH was used as an endogenous reference. Protein expression was quantified as gene grey value/GAPDH grey value. Each analysis was performed in triplicate. The product information of the primary antibodies is listed in Supplementary Table S2.

### 2.8 Flow cytometry

Briefly, the apoptosis rate of the cells prepared from the degenerative and control NP tissues was determined using the Annexin V‐fluorescein isothiocyanate (FITC) apoptosis detection kit (Beyotime, China) according to the manufacturer's instructions.

### 2.9 Co‐immunoprecipitation (Co‐IP)

In brief, whole‐cell extracts were isolated after transfection or stimulation with the appropriate ligands. Then, all samples were initially precleaned with 25 μL of protein A/G‐agarose (50% v/v). Subsequently, the supernatants were immunoprecipitated by incubating with the appropriate antibodies plus protein A/G beads (Santa Cruz Biotechnology, USA) overnight at 4°C. Protein A/G‐agarose‐antigen‐antibody complexes were collected via centrifugation at 12 000 rpm for 60 seconds at 4°C. Beads were washed five times and treated with an IP lysis buffer and separated via SDS–PAGE for Western blot analysis as indicated above.

### 2.10 Ubiquitination analyses

Degenerative or control NP cells that were transfected with siNC or siUSP15 were lysed by sonication with 1% SDS‐containing radioimmunoprecipitation assay (RIPA) buffer on ice. Then, the lysates were treated with Protein A/G PLUS‐Agarose (sc‐2003, Santa Cruz Biotechnology) for 1 hour at 4°C. Thereafter, each sample was incubated with IgG (sc‐2027, Santa Cruz Biotechnology) overnight at 4°C. The anti‐ubiquitin antibody (ab7780, Abcam) was used for immunoblotting.

### 2.11 Statistical analysis

Statistical analyses were performed using the GraphPad Prism software version 7.0 (CA, USA). All data were represented as means ± SEM from three independent experiments. Statistical significance was assessed via one‐way analysis of variance. Differences between the degenerative and control NP samples were accepted as statistically significant at *P* < .05 for all the variables compared.

## 3 RESULTS

### 3.1 USP15 was up‐regulated in degenerative NP tissues or cells

**Figure 1:**
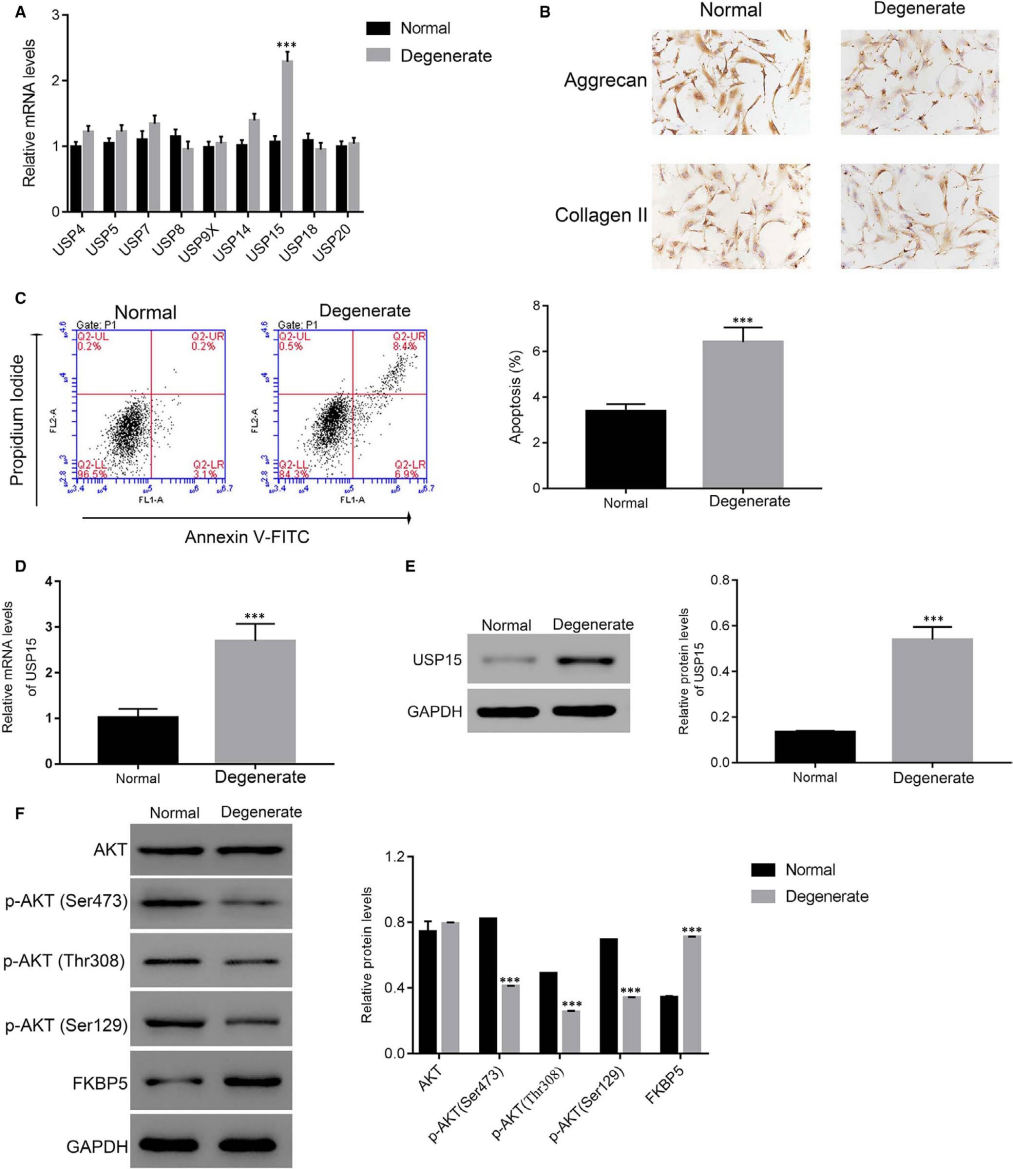
USP15 was up‐regulated in degenerative NP tissues. A, The relative mRNA levels of USP4, USP5, USP7, USP8, USP9X, USP14, USP15, USP18 and USP20 detected in normal and degenerative NP tissues. ***P < .001 vs normal. B, Immunohistochemical analysis was performed to determine the protein levels of aggrecan and collagen II in normal and degenerative NP cells. C, The apoptosis ratio of degenerative NP revealed up‐regulation compared with normal NP cells. ***P < .001 vs normal. D and E, Representative images of the up‐regulation of the relative mRNA and protein levels of USP15 in degenerative NP cells. ***P < .001 vs normal. F. Western blotting was performed to determine the phosphorylation of AKT at different sites and FKBP5 in normal and degenerative NP cells, respectively. ***P < .001 vs normal

We examined the relative mRNA levels of nine members of the DUB family, namely USP4, USP5, USP7, USP8, USP9X, USP14, USP15, USP18 and USP20, in normal (n = 8) and degenerative (n = 10) NP tissues. Interestingly, only USP15 was up‐regulated in degenerative NP tissues compared with in normal tissues. The remaining USP family members showed no significant difference between normal and degenerative NP tissues (Figure 1A).

Immunohistochemical staining was performed to determine the protein levels of aggrecan and collagen II in normal and degenerative NP cells after primary NP cells were isolated from normal and degenerative NP tissues (n = 6). As shown in Figure 1B, both aggrecan and collagen II were expressed in normal and degenerative NP cells. However, the apoptosis rate of degenerative NP cells was much higher than that of normal NP cells (Figure 1C).

Next, we quantified the level of USP15 in normal and degenerative NP cells. Our analyses suggested that the level of USP15 was elevated in degenerative NP cells (Figure 1D,E). Moreover, the protein expression level of FKBP5 was significantly up‐regulated in degenerative NP tissues, whereas p‐AKT at three different sites (Ser473, Thr308 and Ser129) was markedly down‐regulated in degenerative NP tissues compared with in control (Figure 1F).

### 3.2 Silencing and overexpression of USP15 in degenerative NP cells

To further assess the function of USP15, we induced the silencing and overexpression of USP15 in degenerative NP cells. For silencing, siRNAs targeting human USP15 (NM_001252078.1) were synthesized (siUSP15‐1/‐2/‐3) and were used to prepare pLKO.1 constructs. A prepared non‐specific RNA functioned as the NC (siNC). All these constructs were independently transfected into degenerative NP cells. Undoubtedly, both the relative mRNA and protein expression levels of USP15 were markedly suppressed in siUSP15‐transfected cells (Figure S1A,B).

Moreover, significant overexpression at both the mRNA and protein levels was noticeable when the full‐length USP15 cDNA inserted into a lentiviral vector was transiently transfected into degenerative NP cells compared with transfection of the mock recombinant vector NC (oeNC) (Figure S1A,B).

### 3.3 LY294002 reversed the effect of USP15 siRNAs in degenerative NP cells

**Figure 2:**
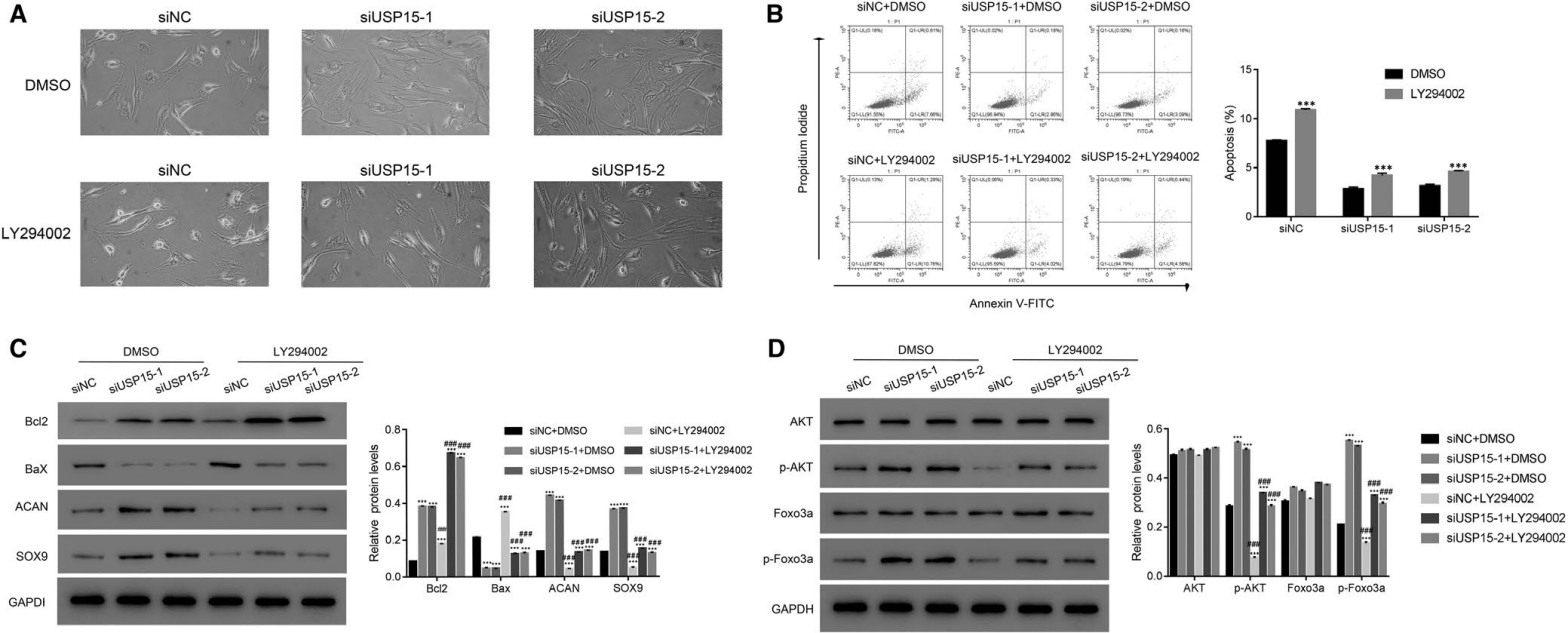
The PI3K inhibitor LY294002 reversed the effect of siUSP15 knockdown in degenerative NP cells. A, Morphology of degenerative NP cells transfected with siNC (control) or siUSP15‐1 (siUSP15‐2) with or without LY294002 treatment. B, The PI3K inhibitor LY294002 improved the apoptosis ratio of siUSP15‐1‐ and siUSP15‐2‐transfected cells. ***P < .001 DMSO. C, The relative protein levels of Bcl2, Bax, ACAN and collagen in siUSP15‐1‐ and siUSP15‐2‐transfected cells with or without LY294002 treatment. ***P < .001 vs siNC + DMSO; ###*P* < .001 vs siUSP15‐1 + DMSO. D, USP15 knockdown of significantly improved the phosphorylation of AKT and Foxo3a in degenerative NP cells. ***P < .001 vs siNC + DMSO; ###*P* < .001 vs siUSP15‐1 + DMSO

First, we analysed the morphology of degenerative NP cells transfected with siNC or siUSP15‐1 (siUSP15‐2) with or without LY294002 treatment. As shown in Figure 2A, the morphology of degenerative NP cells transfected with siNC or siUSP15‐1 (siUSP15‐2) showed no significant differences with or without LY294002 treatment.

Then, we examined the apoptosis rate of siUSP15‐1‐ and siUSP15‐2‐transfected cells. As shown in Figure 2B, the apoptosis rate of siUSP15‐1‐ and siUSP15‐2‐transfected cells was remarkably down‐regulated than that of siNC‐transfected controls. However, the PI3K inhibitor LY294002 significantly rescued USP15 function in degenerative NP cells.

Next, we examined the expression levels of the anti‐apoptotic marker Bcl2 and pro‐apoptotic factor Bax in siUSP15‐1‐ and siUSP15‐2‐transfected NP cells. We observed that USP15 knockdown significantly up‐regulated Bcl2 expression and down‐regulated Bax expression in degenerative NP cells. However, the inhibitor LY294002 disrupted these effects.

ACAN and SOX9 are re‐identified as master chondrogenic transcription factors.^21^ In the present study, USP15 silencing significantly improved the expression levels of ACAN and SOX9 in degenerative NP cells. Meanwhile, these effects were markedly suppressed in the presence of the PI3K inhibitor LY294002 (Figure 2C). Interestingly, USP15 knockdown promoted p‐AKT and Foxo3a phosphorylation (p‐Foxo3a) in degenerative NP cells (Figure 2D).

### 3.4 USP15 function was abolished by the AKT agonist IGF‐1

**Figure 3:**
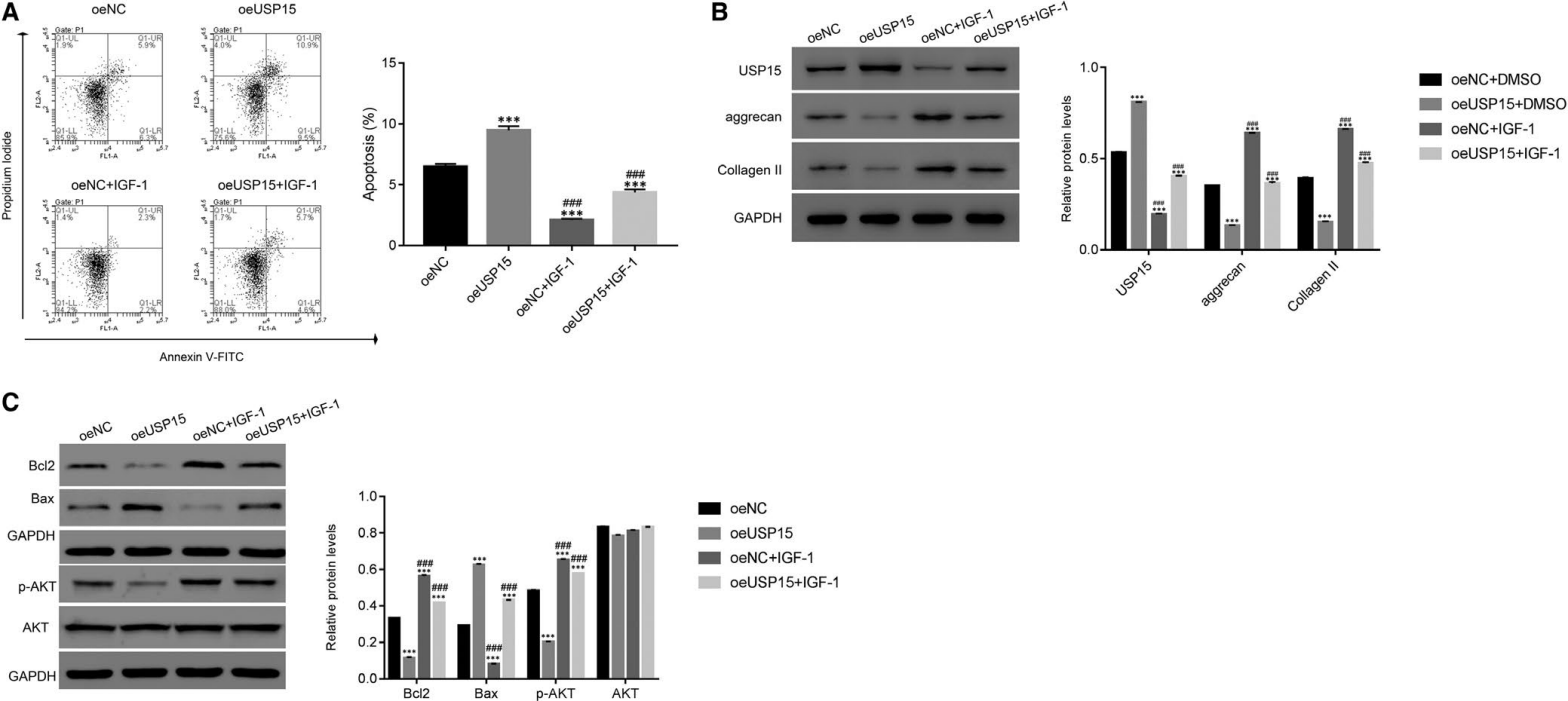
USP15 function of was abolished by the AKT agonist IGF‐1. A, The apoptosis of oeUSP15‐transfected cells was suppressed in the presence of the AKT agonist IGF‐1. ***P < .001 vs oeNC; ###*P* < .001 vs oeUSP15. B, Western blotting was used to examine the protein expression levels of USP15, aggrecan and collagen in oeNC‐ or oeUSP15‐transfected cells treated with the AKT agonist IGF‐1 or untreated. ***P < .001 vs oeNC; ###*P* < .001 vs oeUSP15. C, The protein expression levels of Bcl2, Bax, p‐AKT and AKT were examined in different transfected cells as indicated. ***P < .001 vs oeNC; ###*P* < .001 vs oeUSP15

To further analyse the association between USP15 and AKT, oeUSP15‐transfected cells were cultured in the presence of the AKT agonist IGF‐1 (20 ng/mL). The apoptosis rate of oeUSP15‐transfected cells was markedly increased compared with that of oeNC‐transfected cells. However, the AKT agonist IGF‐1 markedly suppressed the apoptosis rate of oeUSP15‐transfected cells (Figure 3A).

Evidently, oeUSP15 significantly improved USP14 expression in degenerative NP cells, while the AKT agonist IGF‐1 markedly inhibited this function. Moreover, the protein levels of aggrecan and collagen II were substantially reduced in oeUSP15‐transfected cells. However, the AKT agonist IGF‐1 significantly abolished oeUSP15 function in degenerative NP cells (Figure 3B).

Furthermore, USP15 overexpression considerably decreased Bcl2 expression, whereas the AKT agonist IGF‐1 significantly improved Bcl2 expression in oeUSP15‐transfected cells. Meanwhile, the opposite trend was observed for Bax expression in oeUSP15‐transfected cells. Importantly, USP15 overexpression markedly suppressed p‐AKT expression. However, this effect was abrogated by the AKT agonist IGF‐1 in degenerative NP cells (Figure 3C).

### 3.5 miR‐338‐3p mimic suppressed the effects of oeUSP15 in degenerative NP cells

**Figure 4:**
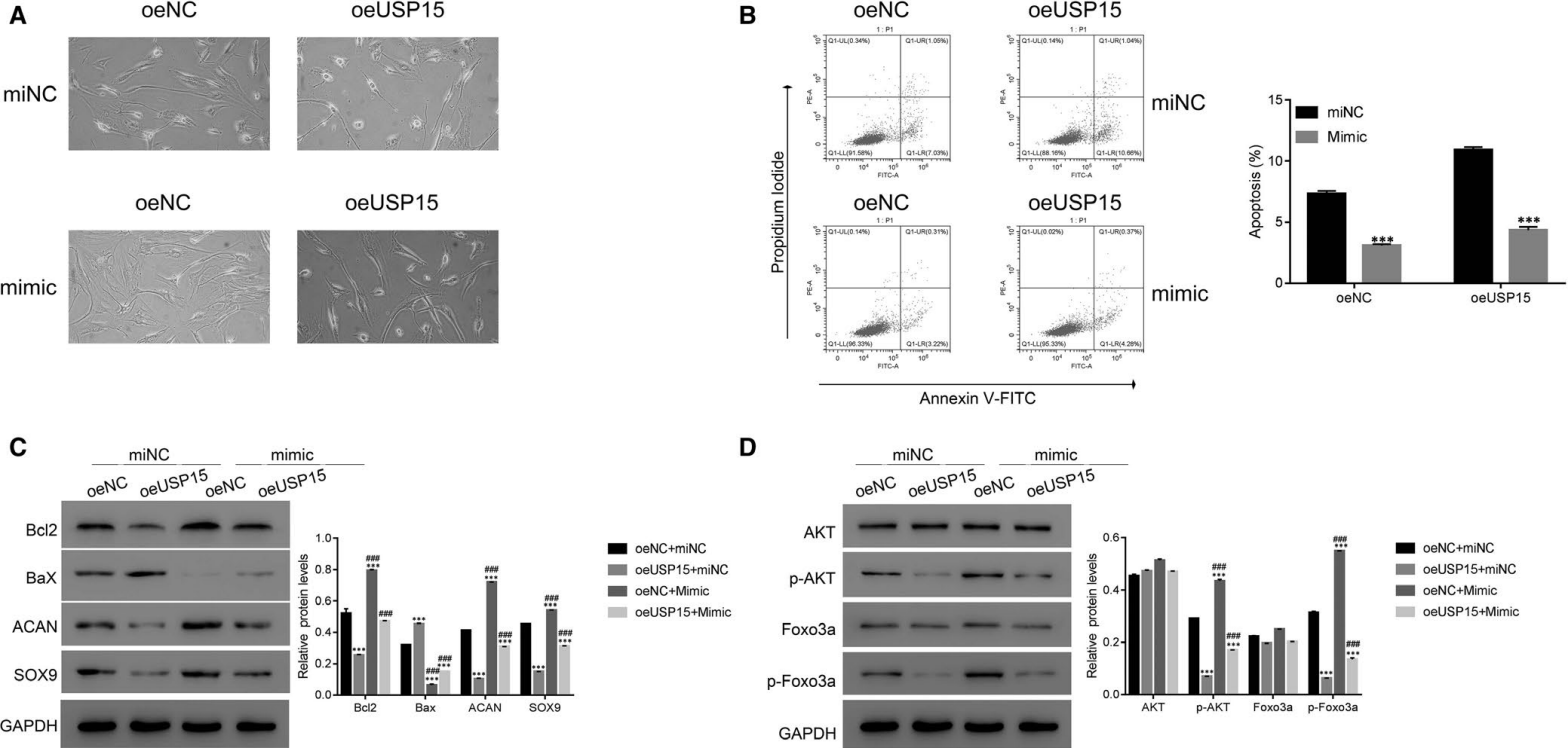
An miR‐338‐3p mimic suppressed the effects of oeUSP15 in degenerative NP cells. A, The morphology of degenerative NP cells transfected with oeNC or oeUSP15‐1 with miNC or mimic treatment. B, The apoptosis of oeNC‐ and oeUSP15‐transfected cells was suppressed in the presence of the miR‐338‐3p mimic. ***P < .001 vs miNC. C and D, Western blotting was performed to determine the protein expression levels of Bcl2, Bax, ACAN, SOX9, AKT, p‐AKT, Foxo3a and p‐Foxo3a in miNC‐ or mimic‐transfected cells as indicated. ***P < .001 vs oeNC + miNC, ###*P* < .001 vs oeUSP15 + miNC

USP15 has been reported to be a target gene of miR‐338‐3p (www.targetscan.org). To analyse the role of miR‐338‐3p in USP15 regulation, we transfected miR‐338‐3p mimic and corresponding miNC into degenerative NP cells transfected with oeNC or oeUSP15, respectively. As shown in Figure 4A, the mimic did not damage the morphology of oeNC‐ or oeUSP15‐1‐transfected degenerative NP cells. Moreover, USP15 overexpression promoted the apoptosis of degenerative NP cells, which was markedly decreased in the presence of the mimic (Figure 4B).

Furthermore, the protein level of Bcl2 was decreased in oeUSP15‐transfected cells and induced with mimic treatment, which was contrary to that observed for Bax. Interestingly, USP15 overexpression suppressed the expression of ACAN and SOX9 in degenerative NP cells, while this effect was markedly inhibited by the mimic (Figure 4C). Our analyses suggest that USP15 overexpression suppresses p‐AKT and p‐Foxo3a. Importantly, our data imply that the mimic contributes to p‐AKT and p‐Foxo3a, its downstream factor, in oeUSP15‐transfected cells (Figure 4D).

### 3.6 miR‐338‐3p binds to the 3′‐ untranslated regions (UTR) of USP15 in degenerative NP cells

**Figure 5:**
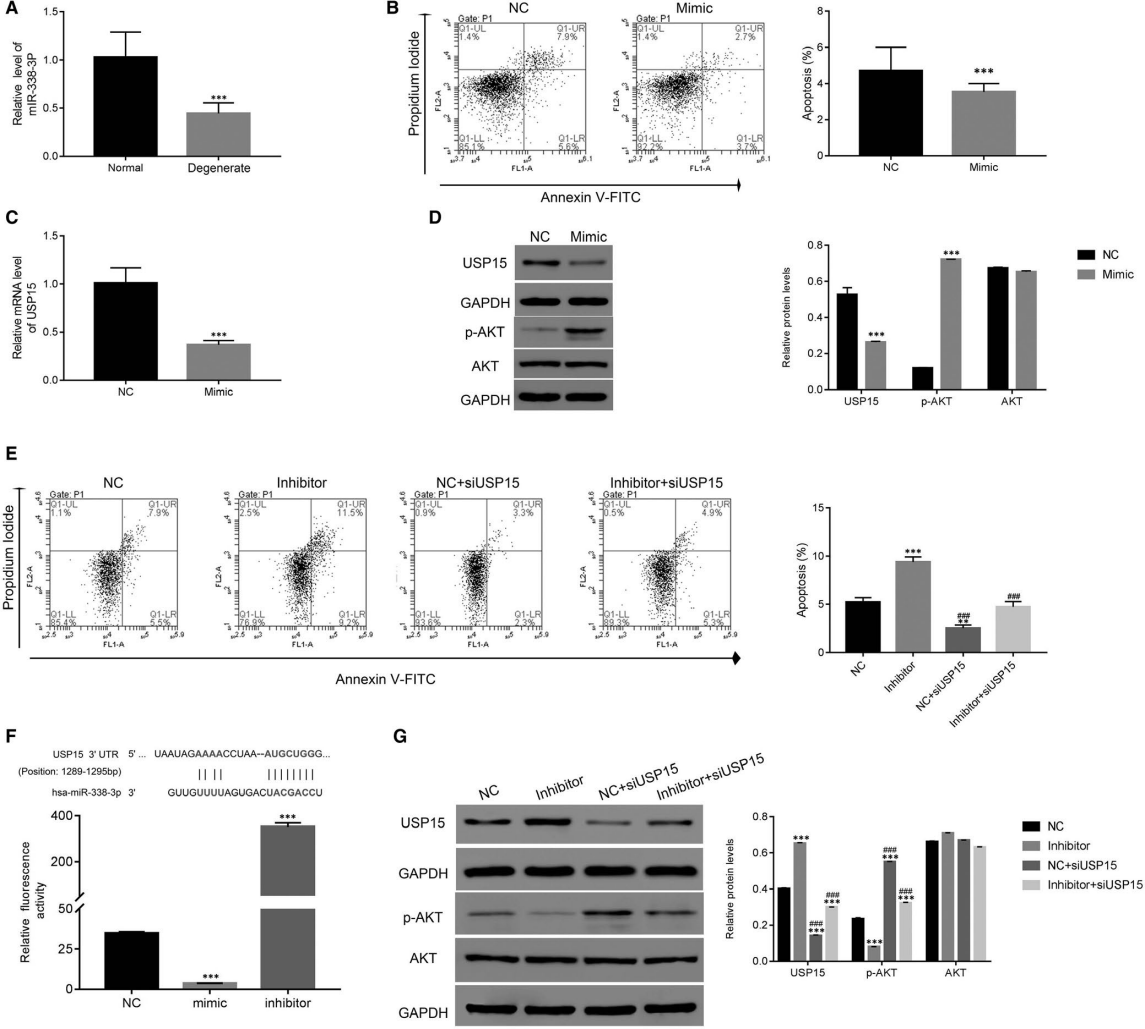
miR‐338‐3p inhibited USP15 function by binding to its 3′UTR. A, miR‐338‐3p was down‐regulated in degenerative NP cells. ***P < .001 vs normal. B, An miR‐338‐3p mimic inhibited apoptosis of degenerative NP cells. ***P < .001 vs NC. C, The relative mRNA level of USP15 was down‐regulated in degenerative NP cells transfected with miR‐338‐3p mimic. ***P < .001 vs NC. D, miR‐338‐3P mimic promoted the phosphorylation of AKT in degenerative NP cells. E, The knockdown of USP15 decreased the apoptosis of degenerative NP cells transfected with the miR‐338‐3p inhibitor. **P < .01 vs NC, ***P < .001 vs NC; ^###^ *P* < .001 vs Inhibitor. F, Dual‐luciferase assays were performed to examine the association between miR‐338‐3p and USP15 in degenerative NP cells. The wild‐type 3′‐UTR of USP15 was cloned into luciferase reporter vectors. Then, degenerative NP cells were transfected with NC, miR‐338‐3p mimics or inhibitor in addition to a recombinant luciferase reporter vector and incubated for 48 h. ***P < .001 vs NC. G, The relative protein levels of USP15, p‐AKT and AKT were examined in NC‐, miR‐338‐3p inhibitor‐, NC + siUSP15‐, miR‐338‐3p inhibitor + siUSP15‐transfected cells. ***P < .001 vs NC; ^###^ *P* < .001 vs inhibitor

Next, we discovered that the level of miR‐338‐3p decreased in degenerative NP tissues (Figure 5A). To further analyse the role of miR‐338‐3p, we transfected miR‐338‐3p mimic and the corresponding NC into degenerative NP cells. As shown in Figure 5B, the miR‐338‐3p mimic significantly suppressed the apoptosis of degenerative NP cells. Interestingly, both the mRNA and protein expression levels of USP15 were markedly decreased in degenerative NP cells after miR‐338‐3p mimic treatment (Figure 5C,D). Significantly, our results indicate that the miR‐338‐3p mimic enhances p‐AKT in degenerative NP cells (Figure 5D).

### 3.7 USP15 knockdown reduced the function of the miR‐338‐3P inhibitor in degenerative NP cells

siUSP15‐transfected cells were co‐cultured with an miR‐338‐3p inhibitor. Undoubtedly, the miR‐338‐3p inhibitor significantly up‐regulated the apoptosis of degenerative NP cells, whereas siUSP15 markedly abolished this function. Therefore, miR‐338‐3p might promote the apoptosis of degenerative NP cells by inhibiting USP15 (Figure 5E).

Furthermore, we assessed the connection between USP15 and miR‐338‐3p by inserting the full length of the USP15 3′‐UTR into the luciferase reporter gene vector. Then, degenerative NP cells were transfected with miR‐338‐3p mimics or inhibitor in addition to the recombined luciferase reporter vector. Obviously, the fluorescence activity of the recombinant vector was significantly down‐regulated in miR‐338‐3p mimic‐transfected cells, whereas it was remarkably improved in miR‐338‐3p inhibitor‐transfected cells (Figure 5F).

Furthermore, we found the miR‐338‐3p inhibitor markedly inhibited p‐AKT. More importantly, siUSP15 significantly up‐regulated p‐AKT in miR‐338‐3p inhibitor‐transfected cells. (Figure 5G).

### 3.8 USP15 interacted with FKBP5 and inhibited its ubiquitination in degenerative NP cells

**Figure 6:**
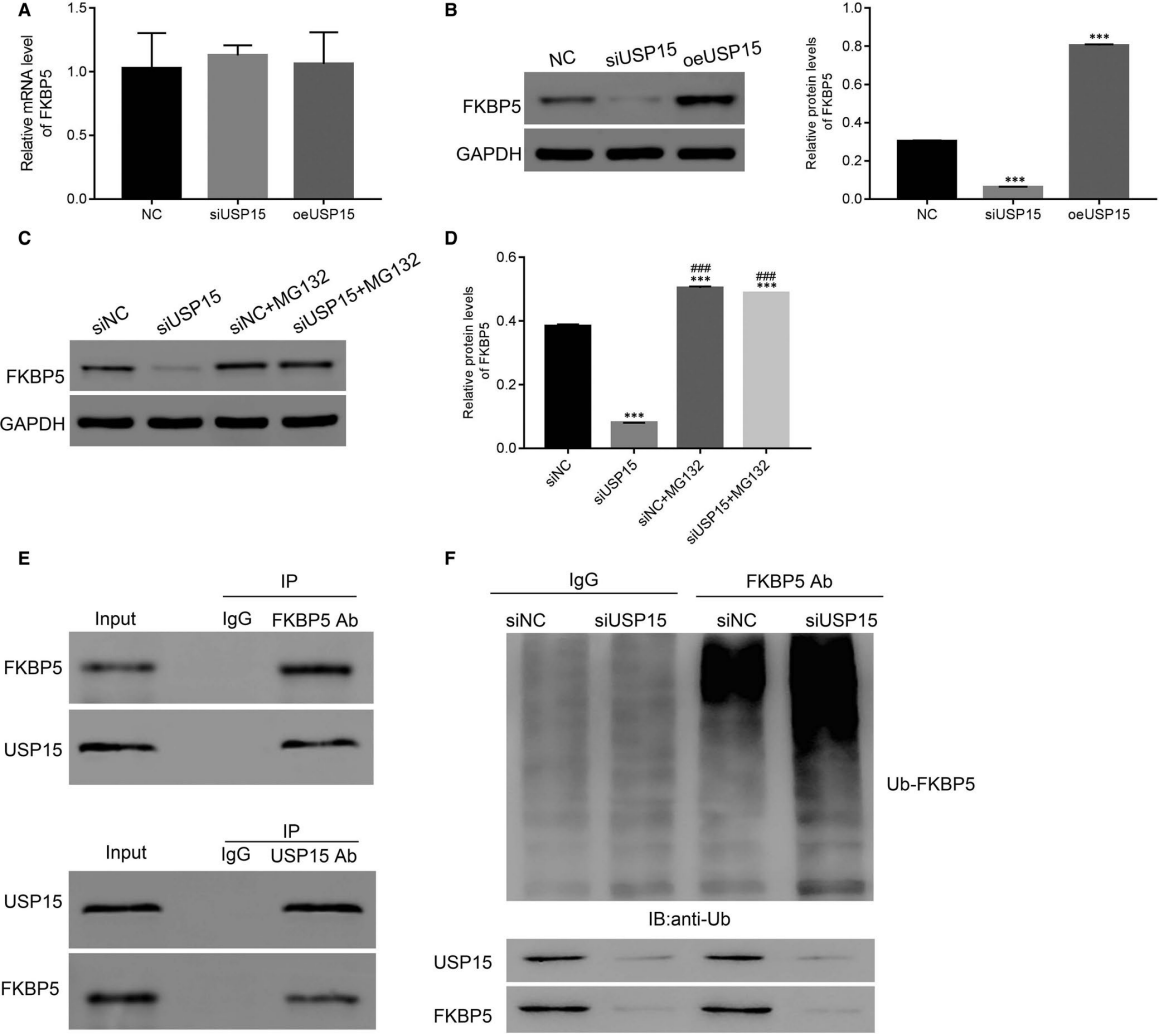
USP15 interacted with FKBP5 and inhibited its ubiquitination in degenerative NP cells. A and B, The relative mRNA and protein levels of FKBP5 were examined in NC‐, siUSP15‐ and oeUSP15‐transfected cells. ***P < .001 vs NC. C and D, The relative protein level of FKBP5 was promoted by MG132 in siUSP15‐transfected cells. E, The co‐IP assay was performed to explore the interaction between USP15 and FKBP5 in degenerative NP cells. F, USP15 knockdown enhanced FKBP5 ubiquitination in degenerative NP cells

Next, we quantified the relative mRNA and protein levels of FKBP5 in siUSP15‐ and oeUSP15‐transfected cells. As shown in Figure 6A, neither siUSP15 nor oeUSP15 disrupted FKBP5 transcription of degenerative NP cells. However, FKBP5 expression was decreased in siUSP15‐transfected cells, whereas it was up‐regulated in oeUSP15‐transfected cells (Figure 6B).

Then, we examined FKBP5 expression in siUSP15‐transfected cells in the presence of the proteasome inhibitor MG132 (10 μM). We found that MG132 significantly promoted FKBP5 expression in siUSP15‐transfected cells (Figure 6C,D).

Finally, the co‐immunoprecipitation (Co‐IP) assay was performed to determine the interaction between FKBP5 and USP15 in degenerative NP cells. Our results demonstrated that there was a stronger interaction between FKBP5 and USP15 in degenerative NP cells (Figure 6E). Moreover, USP15 knockdown enhanced FKBP5 ubiquitination (Ub‐FKBP5) in degenerative NP cells (Figure 6F).

We further explored the relationship between FKBP5 and AKT activity by inducing FKBP5 overexpression in degenerative NP cells (Figure S1C,D) and compared it with mock‐transfected oeFKBP5 counterpart in siUSP15 cells. As shown in Figure S2, both p‐AKT and p‐Foxo3a were significantly improved in siUSP15‐transfected cells, whereas oeFKBP5 transfection markedly suppressed the effect of siUSP15. More importantly, MG132 markedly suppressed p‐AKT and p‐Foxo3a in degenerative cells, as indicated. Therefore, these data suggest that USP15 regulates the activity of the AKT pathway by deubiquitinating and stabilizing FKBP5 in degenerative NP cells.

## 4 DISCUSSION

DDD is a chronic deterioration condition that is mainly initiated within the NP.^22^ The dysfunction of NP cells accelerates the progression of NP degeneration.^3^ However, the underlying molecular mechanism remains unexplored. In the present study, we identified that USP15 was up‐regulated in human degenerative NP tissues and cells. Moreover, our results indicated that USP15 positively correlates with the apoptosis of degenerative NP cells. Therefore, USP15 might be a promising diagnostic marker and therapeutic target of DDD. Our findings not only enhance the understanding of USP15 but also indicate its potential value as a therapeutic target in the prevention of NP degeneration.

It is well‐known that miRNAs binds to the 3′‐UTR to inhibit the transcription of target genes.^23^ Previous studies have demonstrated that miR‐338‐3p can induce the apoptosis of lung cancer and colorectal cancer cells.^24^, ^25^ Furthermore, miR‐338‐3p targets AKT to suppress the proliferation and invasion of lung cancer cells.^26^ In the present study, our findings suggested that miR‐338‐3p was down‐regulated in degenerative NP cells. Meaningfully, our results provided pieces of evidence to indicate that USP15 is the target gene for miR‐338‐3p in degenerative NP cells. Therefore, miR‐338‐3p might suppress the enhanced apoptosis that occurs in degenerative NP cells by targeting USP15.

Collectively, our study is the first to demonstrate that miR‐338‐3p has an anti‐apoptotic function in degenerative NP cells. However, our results showed a discrepancy in the established function of miR‐338‐3p as a tumour suppressor in many types of cancers.^12^, ^13^ Therefore, miR‐338‐3p might present different roles in different tissues. Moreover, our data suggest that miR‐338‐3P inhibitor represses p‐AKT even in the presence of USP15 siRNA, suggesting that miR‐338‐3p regulates p‐AKT at least, in part, independent of USP15 in degenerative NP cells. Therefore, other targets involved in this network are valuable to explore in the following studies.

A previous report has shown that activation of the AKT pathway inhibits the apoptosis of human degenerative disc NP cells.^17^ In this research, our findings suggest that the level of USP15 negatively correlates with the level of p‐AKT. This result indicates that the pro‐apoptosis property of USP15 is attributable to the suppression of the PI3K/AKT pathway in degenerative NP cells. Moreover, the expression of USP15 was suppressed by the PI3K/AKT agonist IGF1 and induced by the inhibitor LY294002 in degenerative NP cells. Therefore, these data illustrate that USP15 mediates the activity of the PI3K/AKT pathway in degenerative NP cells in a negative feedback manner.

In this study, we identified that USP15 interacts with FKBP5 in degenerative NP cells. Additionally, we found that Ub‐FKBP5 is enhanced in siUSP15‐transfected cells. Therefore, USP15 might stabilize FKBP5 expressions in degenerative NP cells via deubiquitination. Furthermore, growing pieces of evidence have demonstrated that FKBP5 down‐regulates p‐AKT and acts as a tumour suppressor.^27^, ^28^ Therefore, USP15 might be a novel component in the FKBP5/AKT signalling pathway in degenerative NP cells.

## CONFLICT OF INTEREST

The authors declare that they have no conflict of interest.

## AUTHOR CONTRIBUTIONS

**Bin Yu:** Data curation (lead). **Bin Shen:** Formal analysis (equal); Software (equal). **Zhaoyu Ba:** Formal analysis (equal); Software (equal). **Zhonghan Liu:** Formal analysis (equal); Software (equal). **Jing Yuan:** Formal analysis (equal); Software (equal). **Weidong Zhao:** Conceptualization (equal); Project administration (equal); Writing‐original draft (equal); Writing‐review & editing (equal). **Desheng Wu:** Conceptualization (lead); Project administration (lead); Writing‐original draft (lead); Writing‐review & editing (lead).

## Supporting information

Fig S1

Fig S2

Supplementary Material

## Data Availability

The data that support the findings of this study are available from the corresponding author upon reasonable request.

## References

[1] Chen D , Xia D , Pan Z , et al. Metformin protects against apoptosis and senescence in nucleus pulposus cells and ameliorates disc degeneration in vivo. Cell Death Dis. 2016;7(10):e2441. 27787519 10.1038/cddis.2016.334 PMC5133996

[2] Kluba T , Niemeyer T , Gaissmaier C , Gründer T . Human anulus fibrosis and nucleus pulposus cells of the intervertebral disc: effect of degeneration and culture system on cell phenotype. Spine. 2005;30(24):2743‐2748. 16371897 10.1097/01.brs.0000192204.89160.6d

[3] Lian C , Gao BO , Wu Z , et al. Collagen type II is downregulated in the degenerative nucleus pulposus and contributes to the degeneration and apoptosis of human nucleus pulposus cells. Mol Med Rep. 2017;16(4):4730‐4736. 28791354 10.3892/mmr.2017.7178 PMC5647025

[4] Antoniou J , Steffen T , Nelson F , et al. The human lumbar intervertebral disc: evidence for changes in the biosynthesis and denaturation of the extracellular matrix with growth, maturation, ageing, and degeneration. J Clin Invest. 1996;98(4):996‐1003. 8770872 10.1172/JCI118884 PMC507515

[5] Sivan SS , Ellen W , Roughley P . Structure, function, aging and turnover of aggrecan in the intervertebral disc. BBA ‐ General Subjects. 2014;1840(10):3181‐3189. 25065289 10.1016/j.bbagen.2014.07.013

[6] Cornelissen T , Haddad D , Wauters F , et al. The deubiquitinase USP15 antagonizes Parkin‐mediated mitochondrial ubiquitination and mitophagy. Hum Mol Genet. 2014;23(19):5227‐5242. 24852371 10.1093/hmg/ddu244 PMC7108632

[7] Pyeon D , Timani KA , Gulraiz F , He JJ , Park IW . Function of ubiquitin (Ub) specific protease 15 (USP15) in HIV‐1 replication and viral protein degradation. Virus Res. 2016;223:161‐169. 27460547 10.1016/j.virusres.2016.07.009 PMC5012541

[8] Zhou L , Jiang H , Du J , et al. USP15 inhibits multiple myeloma cell apoptosis through activating a feedback loop with the transcription factor NF‐κBp65. Exp Mol Med. 2018;50(11):151. 10.1038/s12276-018-0180-4 PMC6244212 30459344

[9] Ouyang ZH , Wang WJ , Yan YG , Wang B , Lv GH . The PI3K/Akt pathway: a critical player in intervertebral disc degeneration. Oncotarget. 2017;8(34):57870–57881. 28915718 10.18632/oncotarget.18628 PMC5593690

[10] Li W , Wu X , Qu R , et al. Ghrelin protects against nucleus pulposus degeneration through inhibition of NF‐κB signaling pathway and activation of Akt signaling pathway. Oncotarget. 2017;8(54):91887‐91901. 29190883 10.18632/oncotarget.19695 PMC5696149

[11] Liu WT , Huang KY , Lu MC , et al. TGF‐|[beta]| upregulates the translation of USP15 via the PI3K|[sol]|AKT pathway to promote p53 stability. Oncogene. 2017;36(19):2715‐2723. 27893708 10.1038/onc.2016.424 PMC5442427

[12] Chen JT , Yao KH , Hua L , Zhang LP , Wang CY , Zhang JJ . miR‐338‐3p inhibits the proliferation and migration of gastric cancer cells by targeting ADAM17. Int J Clin Exp Pathol. 2015;8(9):10922‐10928. 26617808 PMC4637623

[13] Guo B , Liu L , Yao J , et al. miR‐338‐3p suppresses gastric cancer progression through a PTEN‐AKT axis by targeting P‐REX2a. Mole Cancer Res. 2014;12(3):313‐321. 10.1158/1541-7786.MCR-13-0507 24375644

[14] Junmei H , Liewei W . FKBP5 as a selection biomarker for gemcitabine and Akt inhibitors in treatment of pancreatic cancer. PLoS One. 2012;7(5):e36252. 22590527 10.1371/journal.pone.0036252 PMC3348935

[15] Boonying W , Joselin A , Huang E , Qu D , Park DS . Pink1 regulates FKBP5 interaction with AKT/PHLPP and protects neurons from neurotoxin stress induced by MPP+. J Neurochem. 2019;150(3):312‐329. 30734931 10.1111/jnc.14683

[16] Ellsworth KA , Eckloff BW , Li L , et al. Contribution of FKBP5 genetic variation to gemcitabine treatment and survival in pancreatic adenocarcinoma. PLoS One. 2013;8(8):e70216. 23936393 10.1371/journal.pone.0070216 PMC3731355

[17] Wang D , Hu ZM , Hao J , et al. SIRT1 inhibits apoptosis of degenerative human disc nucleus pulposus cells through activation of Akt pathway. Age. 2013;35(5):1741‐1753. 22990594 10.1007/s11357-012-9474-y PMC3776108

[18] Yu S , Yu M , Bu Z , He P , Feng J . FKBP5 exacerbates impairments in cerebral ischemic stroke by inducing autophagy via the AKT/FOXO3 pathway. Front Cell Neurosci. 2020;14(193). 10.3389/fncel.2020.00193 PMC7374263 32760250

[19] Luo K , Li Y , Yin Y , et al. USP49 negatively regulates tumorigenesis and chemoresistance through FKBP51‐AKT signaling. The EMBO J. 2017;36(10):1434‐1446. 28363942 10.15252/embj.201695669 PMC5430216

[20] Das T , Park JK , Park J , et al. USP15 regulates dynamic protein–protein interactions of the spliceosome through deubiquitination of PRP31. Nucleic Acids Res. 2017;45(8):4866‐4880. 28088760 10.1093/nar/gkw1365 PMC5416801

[21] Wei P , Xu Y , Gu Y , Yao Q , Li J , Wang L . IGF‐1‐releasing PLGA nanoparticles modified 3D printed PCL scaffolds for cartilage tissue engineering. Drug Delivery. 2020;27(1):1106‐1114. 32715779 10.1080/10717544.2020.1797239 PMC7470157

[22] Erwin WM , DeSouza L , Funabashi M , et al. The biological basis of degenerative disc disease: proteomic and biomechanical analysis of the canine intervertebral disc. Arthritis Res Ther. 2015;17:240. 26341258 10.1186/s13075-015-0733-z PMC4560915

[23] Zhu J , Yao K , Wang Q , et al. Ischemic postconditioning‐regulated miR‐499 protects the rat heart against ischemia/reperfusion injury by inhibiting apoptosis through PDCD4. Cell Physiol Biochem. 2016;39(6):2364‐2380. 27832626 10.1159/000452506

[24] Zhang G , Zheng H , Zhang G , et al. MicroRNA‐338‐3p suppresses cell proliferation and induces apoptosis of non‐small‐cell lung cancer by targeting sphingosine kinase 2. Cancer Cell Int. 2017;17(1):46. 28428733 10.1186/s12935-017-0415-9 PMC5392967

[25] Lu M , Huang H , Yang J , et al. miR‐338‐3p regulates the proliferation, apoptosis and migration of SW480 cells by targeting MACC1. Exp Ther Med. 2019;17:2807‐2814. 30906469 10.3892/etm.2019.7260 PMC6425231

[26] Liu J , Cao L , Zhao NA , et al. miR‐338‐3p inhibits A549 lung cancer cell proliferation and invasion by targeting AKT and β‐catenin signaling pathways. Mole Med Rep. 2019;20:33‐40. 10.3892/mmr.2019.10215 PMC6579971 31115502

[27] Pei H , Li L , Fridley BL , et al. FKBP51 affects cancer cell response to chemotherapy by negatively regulating Akt. Cancer Cell. 2009;16(3):259‐266. 19732725 10.1016/j.ccr.2009.07.016 PMC2755578

[28] Li L , Fridley BL , Kalari K , et al. Gemcitabine and arabinosylcytosin pharmacogenomics: genome‐wide association and drug response biomarkers. PLoS One. 2012;4(11):e7765. 10.1371/journal.pone.0007765 PMC2770319 19898621

