# Supplementary figures and images for: USP15 promotes the apoptosis of degenerative nucleus pulposus cells by suppressing the PI3K/AKT signalling pathway
Source: J Cell Mol Med. 2020 Nov 1;24(23):13813–23. doi: 10.1111/jcmm.15971 (PMC7754067; doi:10.1111/jcmm.15971)

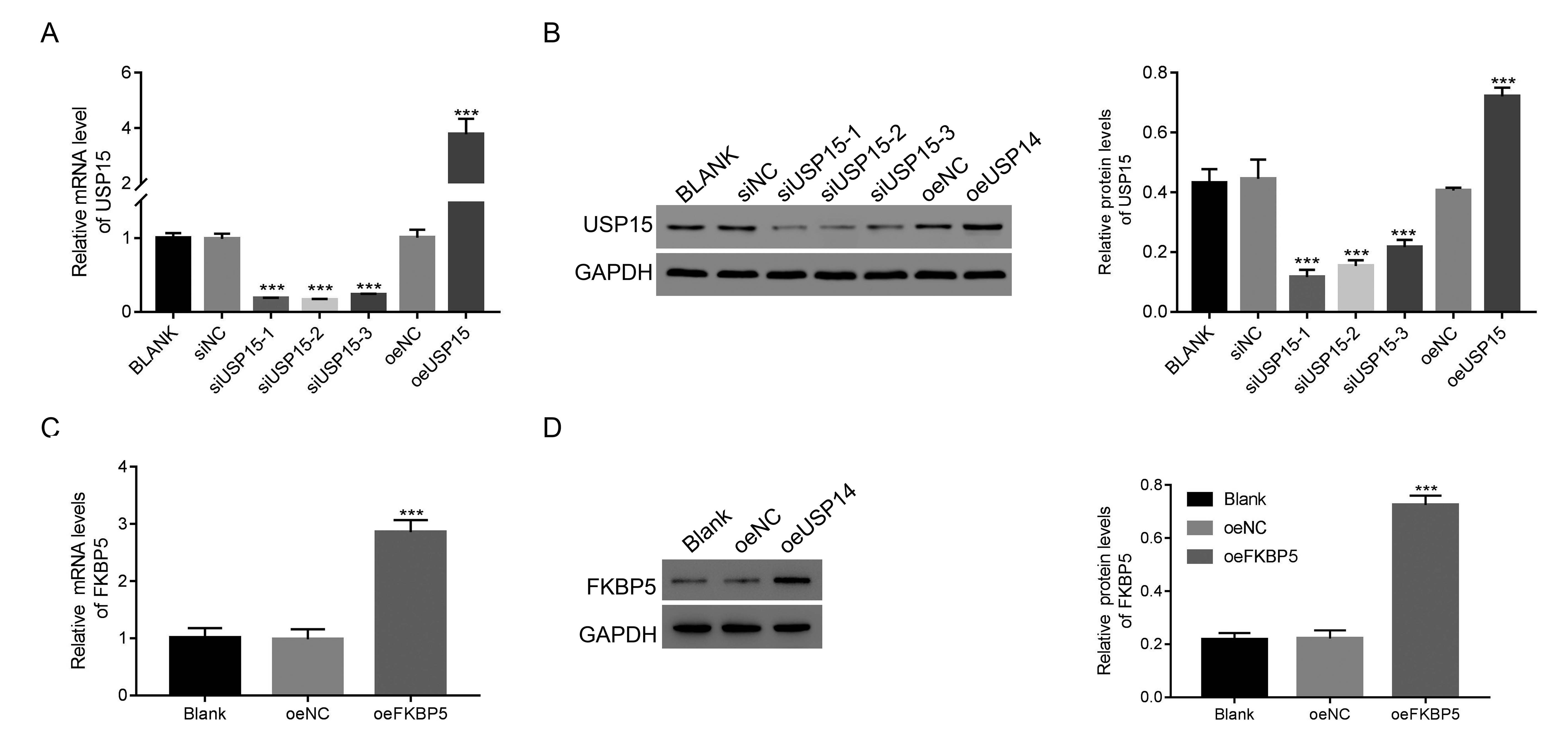

Supplement: Supplementary file 1 — Fig S1 [file JCMM-24-13813-s001.jpg]

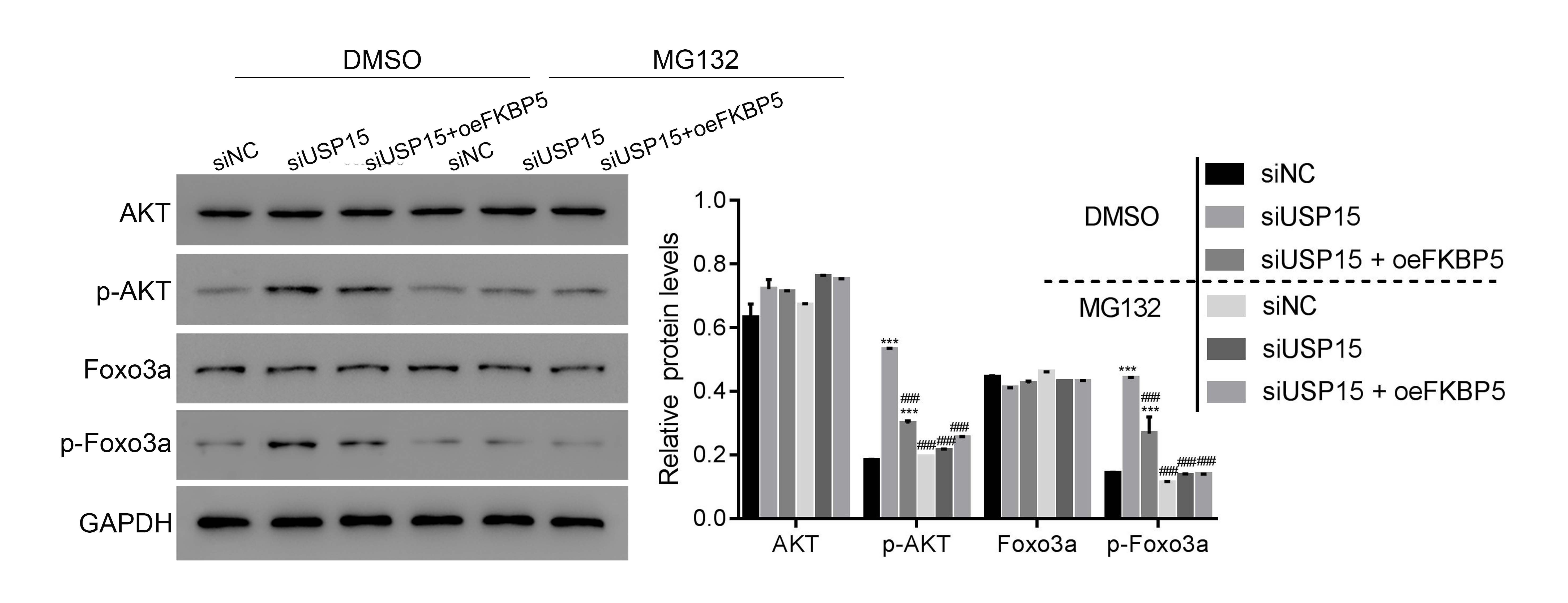

Supplement: Supplementary file 2 — Fig S2 [file JCMM-24-13813-s002.jpg]
