# Supplementary material for: USP15 promotes the apoptosis of degenerative nucleus pulposus cells by suppressing the PI3K/AKT signalling pathway
Source: J Cell Mol Med. 2020 Nov 1;24(23):13813–23. doi: 10.1111/jcmm.15971 (PMC7754067; doi:10.1111/jcmm.15971)
Supplement: Supplementary file 3 — Supplementary Material [file JCMM-24-13813-s003.docx]

**Supplementary File 1: microRNA sequence information**

NC: 5’-CAGUAC UUUUGUGUAGUACAA-3’;

hsa-miR-338-3p: 5’-UCCAGCAUCAGUGAUUUU GUUG-3’;

miR-338-3p inhibitor: 5’-CAACAA AAUCACUGA UGC UGGA-3’;

miR-338-3p mimics: 5’-UCCAGCAUCAGUGAUUUUGUUG-3’.

**Supplementary File2: Primer sequence information**

1.1 Homo sapiens ubiquitin specific peptidase 4 (USP4), transcript variant 3, mRNA

NM_001251877.1

Primer F 5' ACCGAGGCGTGGAATAAAC 3'

Primer R 5' TGGCAACTCAGCACATTGG 3'

Pos:371-543

1.2 Amplified product: Size: 173 bps

Homo sapiens ubiquitin specific peptidase 5 (USP5), transcript variant 1, mRNA

NM_001098536.1

Primer F 5' TTTGCCTCATTCCCTGACTACC 3'

Primer R 5' CGTCTTCGTTGCCATAGAAACC 3'

Pos: 1743- 1974

Amplified product: Size: 232 bps

1.3 Homo sapiens ubiquitin specific peptidase 7 (USP7), transcript variant 2, mRNA

NM_001286457.1

Primer F 5' GAGGCAACCTTTCAGTTC 3'

Primer R 5' CGTGGCATCACCATAATC 3'

Pos: 270- 379

Amplified product: Size: 110 bps

1.4 Homo sapiens ubiquitin specific peptidase 8 (USP8), transcript variant 2, mRNA

NM_001128610.2

Primer F 5' ATCATTCACCCACCAACAC 3'

Primer R 5' AGAAGCAGAAAGCCTTGAG 3'

Pos: 2349- 2623

Amplified product: Size: 275 bps

1.5 Homo sapiens ubiquitin specific peptidase 9 X-linked (USP9X), transcript variant 3, mRNA

NM_001039590.2

Primer F 5' CTTGTCAGCCAGGTGTAGAAG 3'

Primer R 5' GCACTCGGATGATGGATTAGG 3'

Pos: 4151- 4269

Amplified product: Size: 119 bps

1.6 Homo sapiens ubiquitin specific peptidase 14 (USP14), transcript variant 2, mRNA

NM_001037334.1

Primer F 5' GAAAGGAGGAACGCTAAAGG 3'

Primer R 5' CAGGCACAGAACGAATACAC 3'

Pos: 360- 493

Amplified product: Size: 134 bps

1.7 Homo sapiens ubiquitin specific peptidase 15 (USP15), transcript variant 1, mRNA

NM_001252078.1

Primer F 5' TGCCTACTTCCAACTCTC 3'

Primer R 5' GCTCTTCCTTTCCTTCTC 3'

Pos: 6958- 7135

Amplified product: Size: 178 bps

1.8 Homo sapiens ubiquitin specific peptidase 18 (USP18), mRNA

NM_017414.3

Primer F 5' CTCCTTGATTTGCGTTGAC 3'

Primer R 5' TCTTCTTCCCACAGTTCTC 3'

Pos: 860- 1047

Amplified product: Size: 188 bps

1.9 Homo sapiens ubiquitin specific peptidase 20 (USP20), transcript variant 2, mRNA

NM_001008563.4

Primer F 5' GACCTTTGCCCTCACCTTG 3'

Primer R 5' CAGGCGTAACACCACAGTC 3'

Pos: 325- 566

Amplified product: Size: 242 bps

2.0 Homo sapiens FKBP prolyl isomerase 5 (FKBP5), transcript variant 2, mRNA

NM_001145775.2

Primer F 5' CCTTGCTGCCTTTCTGAAC 3'

Primer R 5' ACCCTTGGCTGACTCAAAC 3'

Pos: 1264- 1432

Amplified product: Size: 169

2.1 Homo sapiens glyceraldehyde-3-phosphate dehydrogenase (GAPDH), transcript variant 2, mRNA

NM_001256799.2

Primer F 5' AATCCCATCACCATCTTC 3'

Primer R 5' AGGCTGTTGTCATACTTC 3'

Pos: 436-653

Amplified product: Size: 218 bps

2.2 hsa-miR-338-3p MIMAT0000763

RT-Primer：

5' GTCGTATCCAGTGCAGGGTCCGAGGTATTCGCACTGGATACGACCAACAA 3'

PCR primer：

Primer F 5' CGCGTCCAGCATCAGTGATT 3'

Primer R 5' AGTGCAGGGTCCGAGGTATT 3'

2.3 Homo sapiens RNA, U6 small nuclear 1 (RNU6-1), small nuclear RNA

NR_004394.1

Primer F 5’CTCGCTTCGGCAGCACA3’

Primer R 5’AACGCTTCACGAATTTGCGT3’

Pos: 4-97

Amplified product: Size: 94 bps

**Supplementary** **Table 1: Homo sapiens ubiquitin specific peptidase 15 (USP15), transcript variant 1, mRNA (NM_001252078.1) RNAi targeting locus information**

| RNAi Targeting Locus | | | Sequence |
| --- | --- | --- | --- |
| Name | locus position | |  |
| siUSP15-1 | | 315-333 | GGAACACCTTATTGATGAA |
| siUSP15-2 | | 790-808 | CCAAAGATCTCTCCTTCAT |
| siUSP15-3 | | 1535-1553 | CCAAACCTATGCAGTACAA |

Supplementary Table 2: The primary antibodies information

| Antibody name | Catalog number | Source | Dilution factor |
| --- | --- | --- | --- |
| USP15 | Ab97533 | Abcam, UK | 1:1000 |
| Bcl2 | Sc-492 | Santa, Spain | 1:300 |
| Bax | Sc-493 | Santa, Spain | 1：300 |
| AKT | #9272 | CST, USA | 1:1000 |
| p-AKT | #9271 | CST, USA | 1:1000 |
| FKBP5 | PA1-020 | CST, USA | 1:800 |
| GAPDH | #5174 | CST, USA | 1:2000 |
